# Thermal, dielectrical and mechanical response of α and β-poly(vinilydene fluoride)/Co-MgO nanocomposites

**DOI:** 10.1186/1556-276X-6-257

**Published:** 2011-03-25

**Authors:** Antonio José Paleo, Carlos Martínez-Boubeta, Lluís Balcells, Carlos Miguel Costa, Vitor Sencadas, Senentxu Lanceros-Mendez

**Affiliations:** 1 IPC - Institute for Polymers and Composites, University of Minho, Campus de Azurém, 4800-058 Guimarães, Portugal; 2 IN2UB and Departament d'Electrònica, Universitat de Barcelona, 08028 Barcelona, Spain; 3 ICMAB-CSIC, Campus UAB, 08193 Bellaterra, Spain; 4 CeNTI - Centre for Nanotechnology and Smart Materials, Rua Fernando Mesquita 2785, 4760-034 Vila Nova de Famalicão, Portugal; 5 Center/Department of Physics, University of Minho, Campus de Gualtar, 4710-057 Braga, Portugal

## Abstract

Nanocomposites of the self-forming core-shell Co-MgO nanoparticles, which were of approximately 100 nm in diameter, and poly(vinylidene fluoride) (PVDF) polymer have been prepared. When the polymer is crystallized in the α-phase, the introduction of the nanoparticles leads to nucleation of the γ-phase of PVDF, increasing also the melting temperature of the polymer. With the introduction of the Co-MgO particles, the dielectric constant of the material slightly increases and the storage modulus decreases with respect to the values obtained for the pure polymer.

## Introduction

Nanosized metal and semiconductor particles possess unique electronic, optical, and catalytic properties that are different from those of bulk crystals. These properties make them attractive materials for fabricating hybrid composites based on a polymer matrix with nanoparticle fillers [1], allowing tailor-made electrical and optical composite responses. Among nanoscale fillers, Co and MgO structured particles are particularly promising candidates as they possess a variety of outstanding physicochemical properties which are utilized in the Fischer-Tropsch catalysis, the carbon nanotube synthesis, and for hydrogen storage applications. In addition, the combination of a magnetic-core and dielectric-shell to form a magnetic material with high permeability at high frequency may play an important role in satisfying the requirements for miniaturization of communication equipments [2,3].

Poly(vinylidene fluoride) (PVDF) is the main representative of a family of polymeric materials with interesting scientific and technological properties. For instance, this polymer is known for its outstanding electroactive properties and an unusual high dielectric constant with respect to other polymers [4]. Although the several studies exploring the properties of the polymer nanocomposites as a function of nanosized fillers in PVDF [5], only few studies are focused in systems with core-shell structures embedded in PVDF. For instance, Dang et al. [6] prepared Ag-TiO_2_/PVDF composites for studying the effect of core-shell structure on global dielectric permittivity in the nanocomposites. Petrychuk et al. [7] prepared Fe_3_O_4 _nanoparticles with and without shell of polyaniline dispersed in PVDF, focusing in dispersion and consequently influence in coefficient of squareness of the hysteresis loop and electromagnetic energy absorption.

In this study, nanocomposite films of PVDF in its α and β phases with core-shell Co-MgO nanoparticles were prepared by solution casting technique for different filler concentrations. The influence of the nanoparticles on the polymer phase, degree of crystallinity, mechanical behavior, and dielectric response of the composites was investigated.

## Experimental

Starting powders from Co (Aldrich, 99.8%, Madrid, Spain) and MgO (Alfa Aesar, 99.998%, Ward Hill, USA) were used for producing magnetic Co particles covered with MgO in a single-step process by means of vapor phase condensation [8]. Nanocomposite samples of PVDF (Solef 1010 from Solvay) with magnetic Co-MgO particles were prepared by dispersing the nanoparticles in a solution of PVDF in *N*,*N*-dimethylformamide (DMF, pure grade from Merck, Darmstadt, Germany ). The initial concentration was 1 g of PVDF per mL of DMF. For obtaining a good dispersion, the magnetic nanoparticles within the solution were placed in an ultrasound bath for 8 h. Flexible films were obtained by spreading the solution on a clean glass substrate. Solvent evaporation and polymer crystallization were obtained inside an oven at controlled temperature. The samples in the α-phase were obtained by maintaining the inside an oven for 60 min at 120°C to ensure complete crystallization of the nanocomposite and solvent removal. After the crystallization process, samples were melted at 230°C for 15 min and cooled down at room temperature. Samples in the β-phase were obtained by solvent evaporation at room temperature. The volume fraction of the magnetic Co-MgO nanoparticles was varied from 0.02 to 0.20%.

The characterization of the crystalline phases present in each PVDF sample was achieved by Fourier transform infrared (FTIR) spectroscopy (Perkin-Elmer, model 1610) in attenuated total reflection mode at room temperature. The thermal stability of the nanocomposites was evaluated by means of differential scanning calorimetry (DSC) (Perkin-Elmer Pyris 1) at a heating rate of 10°C/min. Dynamical mechanical analysis (DMA) was carried out at room temperature (Perkin-Elmer DMA-8000), under nitrogen atmosphere. Finally, circular Au electrodes of 5 mm radius were vacuum evaporated onto both sides of each sample to evaluate the dielectric response of the nanocomposites. The real (ε") part of the permittivity and the dielectric losses (tan δ) were obtained in the frequency range 100 Hz to 1 MHz at room temperature with an automatic *Quadtech 1929 *Precision LCR meter.

## Results and discussion

A detailed structural, morphological, and magnetic characterization of the samples has previously been published [8]. Particles of about 100 nm diameter were chosen for this study. The magnesia coating not only protects the high magnetic moment (170 ± 8 emu/g) Co-fcc core from corrosion, but also brings about further improvement by providing polar surfaces for attachment of polymers.

**Figure 1:**
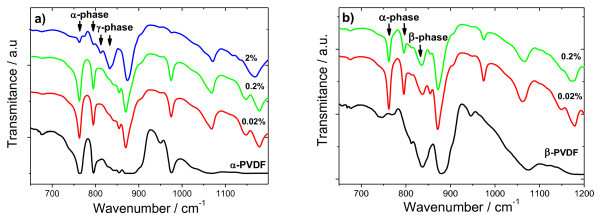
**FTIR spectra of the Co-MgO PVDF nanocomposites crystallized from the melt **(a)**, and crystallized at room temperature **(b)****.

Figure 1a shows FTIR measurements for samples crystallized from the melt. It is observed that increasing the concentration of the nanoparticles induces nucleation of the γ-polymorph [9,10]. In contrast, when the solution crystallizes at room temperature, the electroactive β-phase is obtained; though the presence of nanoparticles modifies the polar crystal PVDF structure, as can be surmised from the presence of α-domains in Figure 1b.

**Figure 2:**
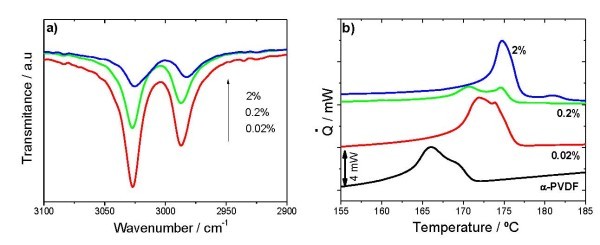
****Infrared spectroscopy and thermal analysis in nanocomposites obtained by crystallization from the melt. (a) ****FTIR absorption band attributed to O-H bonds** (b) **DSC thermograms.

These results suggest that the Co-MgO nanoparticles have an influence on polymer crystallization. A large number of studies have been focused on the crystallization of PVDF resin. For example, addition of hydrate salt Mg(NO_3_)_2 _6H_2_O to PVDF was shown to promote the formation of the β-phase [11]. In the case of this study, hydroxylated MgO surfaces are obtained after exposure of our particles to normal air conditions. Thus, we surmise hydrogen bonding at the interface between PVDF molecules and the Mg-OH pair can preferably lead to better-oriented packing of CH_2_-CF_2 _dipoles, which is the *trans *conformation. We surmise on increasing the particle loading, the stronger polarity of the hydroxyl groups. Evidences are shown in Figure 2a, the broad absorption band attributed to the hydrogen-bonded O-H stretch.

The presence of the different phases and their nature was further confirmed by DSC. Thermograms for samples crystallized from the melt are shown in Figure 2b. The samples containing nanoparticles show two melting peaks. The first one is related to the melting of the α-phase of the polymer, the second one occurring at higher temperatures relates to the melting of the γ-PVDF. It is also observed that the incorporation of the Co-MgO nanoparticles into the polymeric matrix increases its thermal stability, as surmised from the slightly higher melting temperature of the nanocomposite. In this way, a method to obtain γ-PVDF at high or moderate cooling rates from the melt is obtained, without the need to apply thermal annealing at temperatures above 160°C for long term, the usual way to obtain this phase [9].

**Figure 3:**
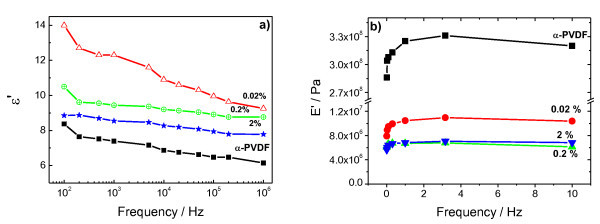
**Electrical and mechanical behavior in nanocomposites obtained by crystallization from the melt. (a) **Dielectric response as a function of frequency and composition** (b)** Dynamical mechanical response

Figure 3a shows the decline of relative dielectric constant according to increasing frequency from 100 Hz to 1 MHz, which revealed the typical model of anomalous dispersion. At a closer inspection, it is demonstrated that incorporating Co-MgO particles increase the dielectric constant for the nanocomposite samples when compared to the pure α-PVDF matrix. The maximum is obtained for the lowest concentration of 0.02 wt.% the decrease in the dielectric constant between 0.2 and 2 wt.% loadings may be a signature of dipolar interactions influencing the nanoparticles intrinsic magnetic properties [12].

For samples crystallized at room temperature (data not shown), the dielectric response of the polymer is quite similar, and shows that the nanoparticles do not improve the dielectric response of the β-PVDF. With respect to the dynamical mechanical response, on the other hand, the pure α-phase matrix has highest mechanical properties when compared with the nanocomposite samples (Figure 3b), which can be related to defect formation around the nanoparticles, due to the interaction with the nanoparticle shell, and a decrease in the degree of crystallinity as observed in the DSC scans.

## Conclusions

The introduction of Co-MgO core-shell fillers within a PVDF matrix influences the polymer crystallization. A stable MgO oxide shell protected the magnetic metallic Co core from oxidation to realize a high saturation magnetization, while altering the PVDF molecules packaging via Mg-OH bonding at the polymer/particle interface. The dielectric properties are slightly affected but, on the other hand, the storage modulus is strongly reduced.

## Abbreviations

DMF: *N*,*N*-dimethylformamide; FTIR: Fourier transform infrared; PVDF: poly(vinylidene fluoride).

## Competing interests

The authors declare that they have no competing interests.

## Authors' contributions

SLM proposed the research work, coordinate the collaborations and carried out the analysis and interpretation of the experimental results. AJP, CMC and VS participated in sample processing, experimental measurements, analysis and interpretation of the results. CBM and LLB produced the nanofillers and carried out the TEM characterization of the nanoparticles. All authors read and approved the final manuscript.
